# Rhythmic expression of the melatonergic biosynthetic pathway and its differential modulation *in vitro* by LPS and IL10 in bone marrow and spleen

**DOI:** 10.1038/s41598-020-61652-5

**Published:** 2020-03-16

**Authors:** Marlina O. Córdoba-Moreno, Ewerton da Silva de Souza, Caroline L. Quiles, Débora dos Santos-Silva, Gabriela S. Kinker, Sandra M. Muxel, Regina P. Markus, Pedro A. Fernandes

**Affiliations:** 1 Laboratory of Neuroimmunoendocrinology, Department of Physiology, Institute of Bioscience, University of São Paulo, 05508-900 São Paulo, Brazil; 2 Laboratory of Chronopharmacology, Department of Physiology, Institute of Bioscience, University of São Paulo, 05508-900 São Paulo, Brazil; 3 Laboratory of Trypanosomatid Physiology, Department of Physiology, Institute of Bioscience, University of São Paulo, 05508-900 São Paulo, Brazil

**Keywords:** Circadian rhythms, Bone marrow, Spleen

## Abstract

Daily oscillation of the immune system follows the central biological clock outputs control such as melatonin produced by the pineal gland. Despite the literature showing that melatonin is also synthesized by macrophages and T lymphocytes, no information is available regarding the temporal profile of the melatonergic system of immune cells and organs in steady-state. Here, the expression of the enzymes arylalkylamine-N-acetyltransferase (AA-NAT), its phosphorylated form (P-AA-NAT) and acetylserotonin-O-methyltransferase (ASMT) were evaluated in phagocytes and T cells of the bone marrow (BM) and spleen. We also determined how the melatonergic system of these cells is modulated by LPS and the cytokine IL-10. The expression of the melatonergic enzymes showed daily rhythms in BM and spleen cells. Melatonin rhythm in the BM, but not in the spleen, follows P-AA-NAT daily variation. In BM cells, LPS and IL10 induced an increase in melatonin levels associated with the increased expressions of P-AA-NAT and ASMT. In spleen cells, LPS induced an increase in the expression of P-AA-NAT but not of melatonin. Conversely, IL10 induced a significant increase in melatonin production associated with increased AA-NAT/P-AA-NAT expressions. In conclusion, BM and spleen cells present different profiles of circadian production of local melatonin and responses to immune signals.

## Introduction

Organs and cells of the immune system present daily variations regulated by oscillators present in each cell^1–6^. The intrinsic circadian clock of most of the immune cells imposes circadian expression of downstream genes and functions^4^. This is the case for the expression of pattern-recognition receptors and cytokines, the recruitment to tissues and the phagocytic activity of monocytes, macrophages and microglia^7–10^. Clock genes are also circadian expressed in mouse lymph nodes^10,11^ and in B splenic cells^12^, where they control the activity of the cells^4,11^. Besides the intrinsic rhythmicity of cells and organs, there is a central synchronization that relies on neural and hormonal signaling controlled by the central clock in the suprachiasmatic nuclei^13,14^. After a sympathetic input, the darkness hormone melatonin, prolactin and glucocorticoids impose, for example, a daily rhythm in the migration of leukocytes to peripheral tissues^6^.

In vertebrates, melatonin is known to be produced in a rhythmic manner by the pineal gland and retina, constitutively by the gastrointestinal tract and on demand by some immunocompetent cells^15,16^. Activated monocytes/macrophages/microglia and T lymphocytes expressed the enzymes arylalkylamine-N-acetyltransferase (AA-NAT), its active phosphorylated form (P-AA-NAT) and acetylserotonin-O-methyltransferase (ASMT) and melatonin^17–21^. In the spleen and in the bone marrow (BM), some works have shown the expression and activity of the melatonergic enzymes^22–24^, however, whether the immune cells of these organs also present circadian variations of the melatonergic system in normal conditions was not explored in a systematic manner. Interestingly, in the bone marrow (BM), for example, some evidences suggest that the melatonin, produced locally at night, increases the proliferation and retention of long-term hematopoietic stem cells, being a key player in maintaining the pool of the most primitive stem cells^25^.

Over the last decades our group have described the Immune-Pineal Axis^26^. During immune responses, pro-inflammatory signals such as LPS and TNF reduce the production of melatonin by the pineal gland^27–31^. Considering that melatonin reduces the migration of immune cells trough the endothelial layer^29,32^, the reduction of melatonin production by the pineal gland is essential for the appropriate mounting of an immune response. Interestingly, TNF and LPS increase the production of melatonin by linage and colostrum macrophages^17,21,33^ and the production of melatonin by immune competent cells seems to be relevant to several immune functions, such as phagocytosis^34^. Others immune signals like IFN-ϒ, glucocorticoids and high levels of ATP also impose dual effects on the pineal gland and immune cells^35–38^. Although the production of melatonin by the BM seems to be dependent on TNF^25^, the responsiveness of the melatonergic system to immune signals, to the best of our knowledge, have never been evaluated in spleen and BM total cells.

The aim of the present work is to evaluate the levels of melatonin and the expression of the melatonergic biosynthetic enzymes AA-NAT, P-AA-NAT and ASMT in monocytes/macrophages and T lymphocytes from the spleen and the BM of rats. Our hypothesis was that the melatonergic system, as observed for several immune pathways, present daily variations in the cells of these two important lymphoid organs of the immune system, and that the BM is able to produce its own melatonin. Moreover, we also aimed to evaluate how the melatonergic system of BM and spleen cells is altered by pro and anti-inflammatory signals.

## Results

### Melatonin production and expression of melatonergic enzymes in the BM and the spleen

**Figure 1:**
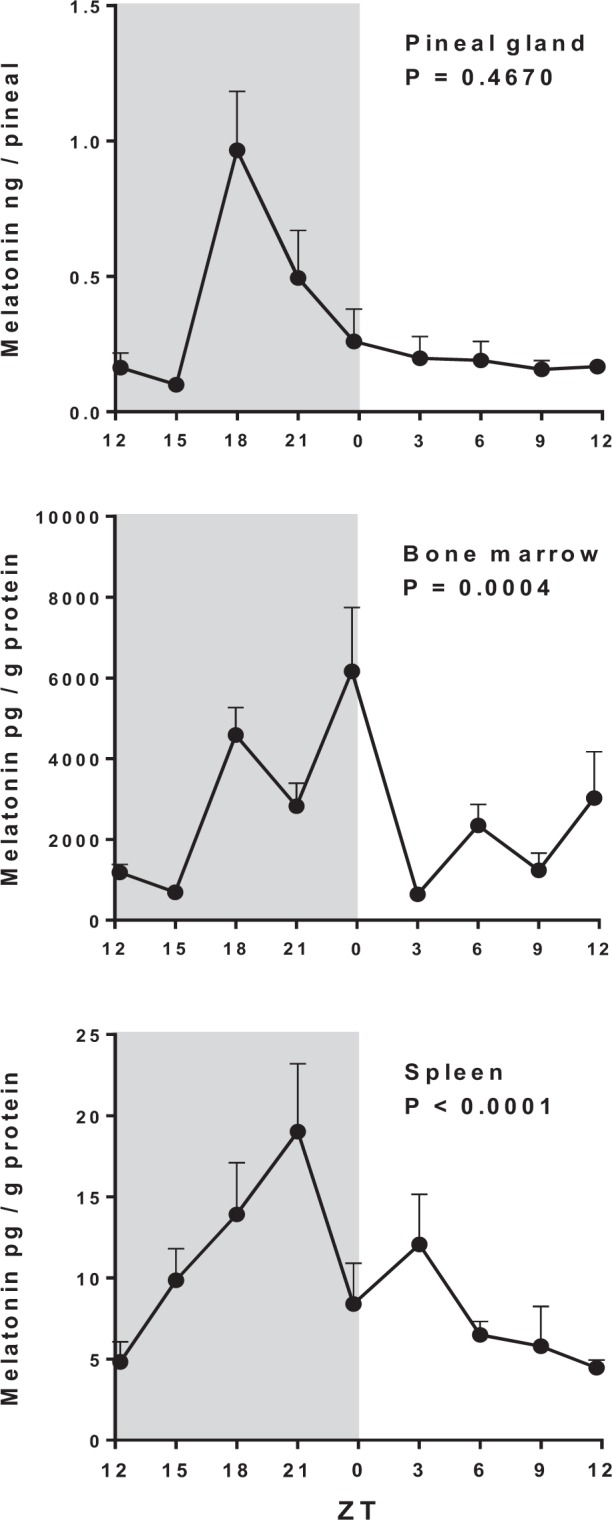
Daily melatonin levels in the pineal gland, the bone marrow and the spleen. Melatonin was determined by HPLC in the pineal gland or by ELISA in the bone marrow and the spleen. ZT0 being defined as the moment when lights went on and ZT12 when the lights are turned off. Results are expressed as the mean ± SEM (n = 3–5 animals per point). Data were analyzed using a Cosinor analysis to determine if the variations have a circadian rhythm. Gray background marks the dark period.

The melatonin levels in the BM and the spleen were evaluated at nine different Zeitgeber times (ZT). The content of melatonin in both organs follow a circadian profile (Supplementary Table 1), and the content in the BM was 1000 fold higher than in the spleen (Fig. 1). In order to evaluate whether the BM and the spleen could synthesize melatonin, we determined the expression of the enzymes AA-NAT, its active form (P-AA-NAT), and ASMT.

**Figure 2:**
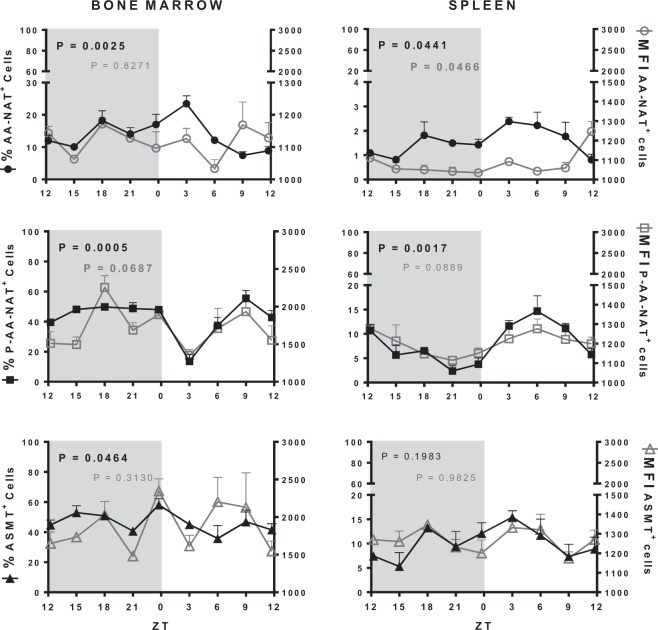
Melatonergic biosynthetic pathway expression in the bone marrow and in the spleen. Left Y axis (full shapes): percentage of cells expressing AA-NAT, P-AA-NAT or ASMT; right Y axis (empty shapes): MIF of the AA-NAT, P-AA-NAT or ASMT positive cells. ZT0 being defined as the moment when lights went on and ZT12 when the lights are turned off. Results are expressed as the mean ± SEM (n = 3 animals per point). Data were analyzed using a Cosinor analysis to determine if the variations have a circadian rhythm. Gray background marks the dark period.

In the BM, the percentage of cells expressing AA-NAT and P-AA-NAT had complementary profiles and presented circadian rhythms, with maximal and minimal at ZT03, respectively. The mean fluorescence intensity (MFI) of AA-NAT and P-AA-NAT had non-circadian rhythms, but the MFI of P-AA-NAT peaked at ZT18. Thus, the active form of the enzyme that converts serotonin in N-acetylserotonin is available at nighttime. ASMT positive cells showed rhythmic expressions (Fig. 2, Supplementary Table 2). In the context of the BM, it is also relevant to mention that around 40–60% of the cells express P-AA-NAT and/or ASMT.

In the spleen, less than 20% of the cells expressed the enzymes of synthesis of melatonin. The percentage of cells expressing AA-NAT and P-AA-NAT, as well as the MFI of AA-NAT followed a circadian rhythm, with higher expression at daytime. However, neither the MFI of P-AA-NAT nor ASMT presented a rhythmic variation (Fig. 2, Supplementary Table 2), strongly suggesting that it does not contributes to daily melatonin rhythm in the spleen.

### Characterization of monocytic, lymphocytic and other lineages in the BM and spleen

As we know, several studies have already shown that monocytes and T lymphocytic cells can produce melatonin, and it is possible that these cells are contributing to the levels of melatonin observed in the organs. Therefore, before looking at its expression profile of melatonin enzymes, we wanted to characterize the percentage of monocytes/macrophages/neutrophils (CD11b^+^), T lymphocytes (CD3^+^) and other cells (CD11b^−^/CD3^−^) in the BM and spleen, since this distribution is specific for each organ.

**Figure 3:**
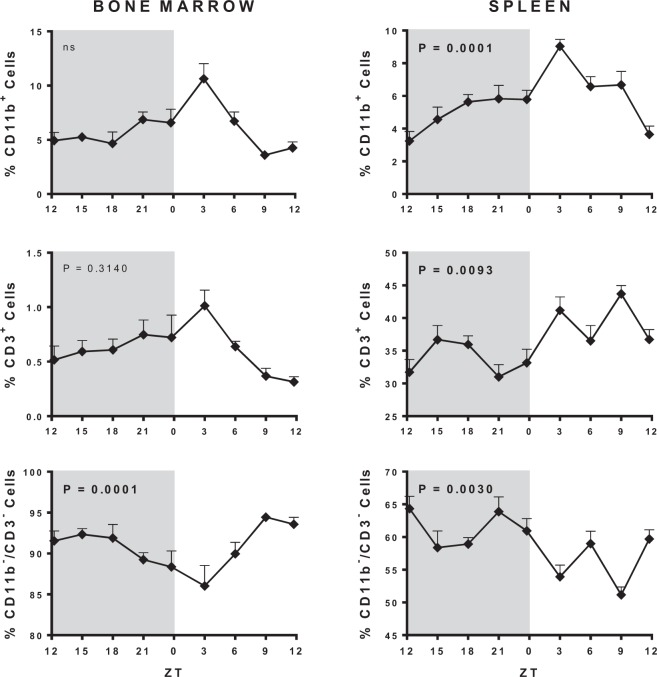
Daily cellular variation in the bone marrow and the spleen. ZT0 being defined as the moment when lights went on and ZT12 when the lights are turned off. Results are expressed as the mean ± SEM (n = 3 animals per point). Data were analyzed using a Cosinor analysis to determine if the variations have a circadian rhythm. Gray background marks the dark period.

In the BM most of the cells are CD11b^−^/CD3^−^ (85–95%) and their percentages followed a circadian rhythm. On the other hand, CD11b^+^ (5–10%) and CD3^+^ (0.5–1%) did not follow rhythmic profiles (Fig. 3, Supplementary Table 1). In the spleen, the percentage of CD11b^−^/CD3^−^ reached 50–65%, while CD3^+^ mount up to 30–45%, and CD11b^+^ up to 3–10%. All three categories of cells follow a circadian rhythm (Fig. 3, Supplementary Table 1). Thus, the proportion of CD11b^−^/CD3^−^ cells is much higher in the BM than in the spleen, while the proportion of CD3^+^ is higher in the spleen when compared to the BM, and the daily variation of the CD3^+^ cells was more prominent in the spleen than in the BM.

### Biosynthetic pathway in the BM and the spleen specific cells

The expression of AA-NAT, P-AA-NAT and ASMT was evaluated according to the percentage of cells expressing the enzyme in each cell category as well as the MFI in the positive cells, which reflects the amount of enzyme expressed. Considering that the synthesis of melatonin is directly dependent on the presence of P-AA-NAT and ASMT, it is important to evaluate whether the rhythm of these enzymes is in phase or out of phase.

**Figure 4:**
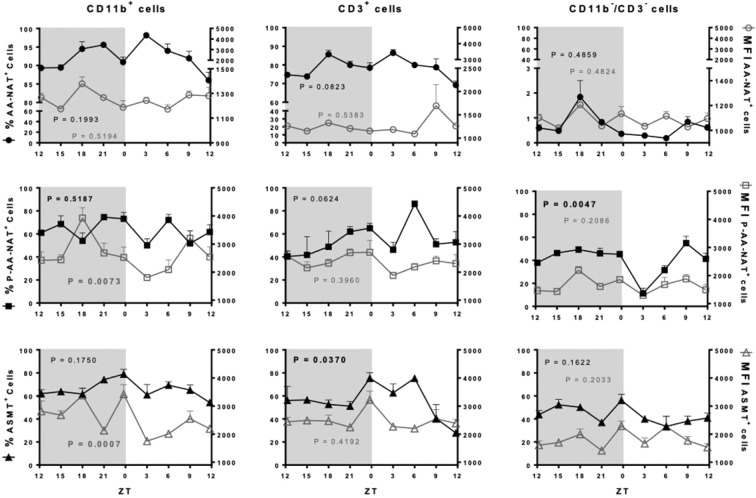
Melatonergic biosynthetic pathway expression in the CD11b^+^, CD3^+^ and CD11b^−^/CD3^−^ bone marrow cells. Left Y axis (full shapes): percentage of cells expressing AA-NAT, P-AA-NAT or ASMT; right Y axis (empty shapes): MFI of the AA-NAT, P-AA-NAT or ASMT positive cells. ZT0 being defined as the moment when lights went on and ZT12 when the lights are turned off. Results are expressed as the mean ± SEM (n = 3 animals per point). Data were analyzed using a Cosinor analysis to determine if the variations have a circadian rhythm. Gray background marks the dark period.

In the BM, 70% of cells expressing AA-NAT are CD11b^+^ and CD3^+^, while only 3% are CD3^−^/CD11b^−^ cells. Fourier and Cosinor analyses showed that neither the percentage of cells nor the MFI expressing AA-NAT presented a circadian rhythm. Otherwise, the active enzyme P-AA-NAT and ASMT were highly expressed (at least 40%) in the three categories of cells, with a circadian rhythm observed only in the percentage of CD11b^−^/CD3^−^ and CD3^+^ cells expressing P-AA-NAT and ASMT, respectively. In the three cell types analyzed, the MFI of P-AA-NAT and ASMT were higher compared to AA-NAT. The MFI of P-AA-NAT followed a circadian rhythm in the CD11b^+^ cells and presented a rhythmic profile in the CD11b^−^/CD3^−^ cells (Fig. 4, Supplementary Table 2).

**Figure 5:**
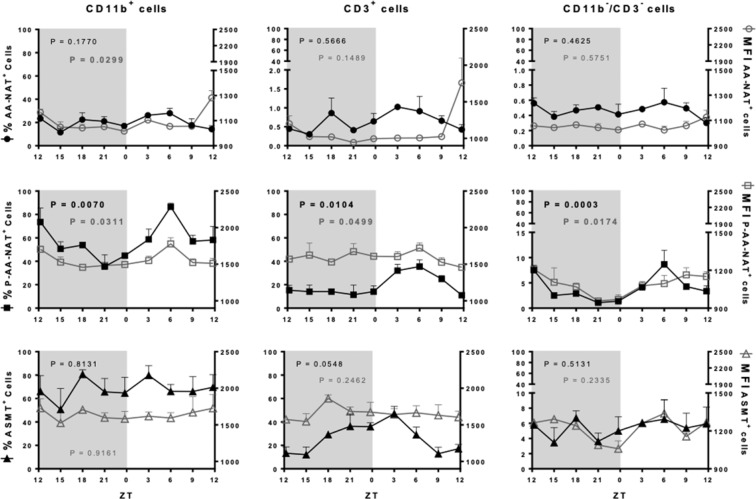
Melatonergic biosynthetic pathway expression in the CD11b^+^, CD3^+^ and CD11b^−^/CD3^−^ splenic cells. Left Y axis (full shapes): percentage of cells expressing AA-NAT, P-AA-NAT or ASMT; right Y axis (empty shapes): MFI of the AA-NAT, P-AA-NAT or ASMT positive cells. Samples were collected each 3 hours. ZT0 being defined as the moment when lights went on and ZT12 when the lights are turned off. Results are expressed as the mean ± SEM (n = 3 animals per point). Data were analyzed using a Cosinor analysis to determine if the variations have a circadian rhythm. Gray background marks the dark period.

A different profile was observed for the spleen cells. In spite of only 1% of CD3^+^ cells expressed AA-NAT, 20–40% of these cells expressed P-AA-NAT and/or ASMT, strongly suggesting that the cells were instrumented to synthesize melatonin. Regarding the monocytic lineage (CD11b^+^), around 30% of the cells expressed AA-NAT and 40–80% expressed P-AA-NAT and/or ASMT, while CD11b^−^/CD3^−^ cells almost did not express the melatonergic synthetic enzymes (Fig. 5). The expression of P-AA-NAT in the three cell types evaluated and the MFI of AA-NAT in the CD11b^+^ cells, presented circadian rhythms (Supplementary Table 2).

### Correlation between melatonergic enzymes and local melatonin content

In order to access whether the melatonin found in BM and spleen is being produced locally, Pearson correlation was performed to compare the levels of melatonin with the expression of the melatonergic biosynthetic pathway enzymes (MFIs) and the percentage of cells expressing the enzymes. In addition, as those parameters indirectly measure the amount of protein expressed in each cellular population, the ZTs when more enzymes are globally being expressed in the cells are the moments where we expected to find higher melatonin levels. Therefore, we created predictive indexes of melatonin synthesis by summing either the MFIs or the percentage of P-AA-NAT and ASMT in each ZTs for total cells and each cell subtype. We then correlated such values with the levels of melatonin detected in the tissues.

**Figure 6:**
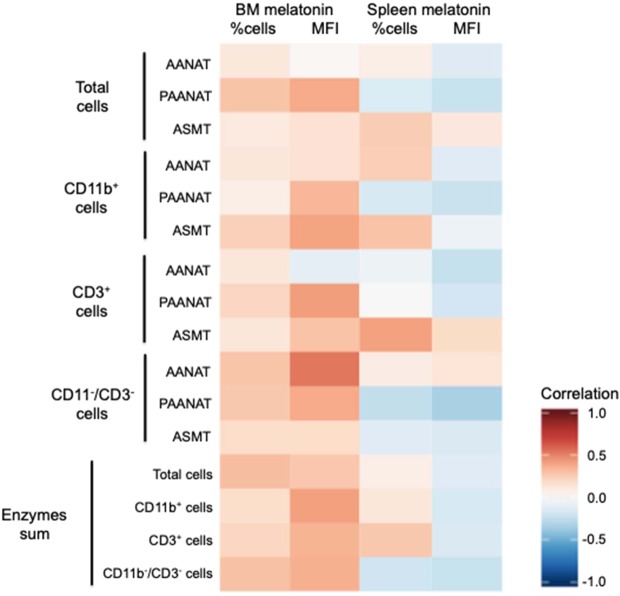
Heatmap correlation between the melatonin levels and the expression of the melatonergic biosynthetic pathway enzymes in the bone marrow and the spleen. The heatmap was performed with the r coefficients of the Pearson correlations in R program. Enzymes sum correspond to the sum of the percentage of cell or the MFI of the melatonergic enzymes (P-AA-NAT and ASMT) in each cellular population. Data for the Pearson correlation (r coefficient and P-value) are showed in Table 1.

In the BM, several of the correlations between melatonin and the MFIs of the enzymes (separated or summed) for total cells and the different cellular populations were positive (Fig. 6, Table 1). Among the enzymes, the correlation of P-AA-NAT expression and melatonin was consistently significant in total cells and in all cell populations. ASMT and AA-NAT expressions were also significantly correlated with melatonin in CD11b^+^ and CD11^−^/CD3^−^ cells, respectively. Accordingly, the variations observed throughout the day in the levels of melatonin and in MFI index display a similar pattern (Supplementary Fig. 1). Finally, the correlations between melatonin and the frequency of cells expressing the enzymes (separated or summed) were not significant in any case (Fig. 6, Table 1). In the spleen, only one significant positive correlation was seen between the local melatonin and the percentage of cells expressing ASMT in CD3^+^ cells (Fig. 6, Table 1).

**Table 1:** Pearson correlation between the melatonin levels and the melatonergic biosynthetic pathway enzymes.

		Bone marrow melatonin				Spleen melatonin			
		% Cells		MFI		% Cells		MFI	
		r	P-value	r	P-value	r	P-value	r	P-value
Total cells	AA-NAT	0,1101	0,2922	0,0227	0,4552	0,0701	0,3642	−0,1440	0,2369
	P-AA-NAT	0,2865	0,0737	0,3804	0,0251	−0,1470	0,2321	−0,2319	0,1222
	ASMT	0,1080	0,2960	0,1508	0,2264	0,2551	0,0996	0,1111	0,2907
CD11b^+^ cells	AA-NAT	0,1218	0,2726	0,1550	0,2201	0,2499	0,1044	−0,1265	0,2648
	P-AA-NAT	0,0686	0,3670	0,3345	0,0441	−0,1714	0,1963	−0,2254	0,1291
	ASMT	0,2389	0,1150	0,4061	0,0178	0,2933	0,0688	−0,0581	0,3867
CD3^+^ cells	AA-NAT	0,1205	0,2747	−0,1004	0,3092	−0,0515	0,3994	−0,2372	0,1168
	P-AA-NAT	0,2193	0,1359	0,4201	0,0146	0,0095	0,4813	−0,1857	0,1769
	ASMT	0,1261	0,2655	0,2932	0,0688	0,4109	0,0166	0,1995	0,1592
CD11b^−^/CD3^−^ cells	AA-NAT	0,2824	0,0768	0,5361	0,0020	0,0889	0,3297	0,1385	0,2454
	P-AA-NAT	0,2714	0,0854	0,3797	0,0254	−0,2482	0,1059	−0,3304	0,0462
	ASMT	0,1897	0,1716	0,1946	0,1654	−0,1196	0,2762	−0,1554	0,2194
Sum	Total cells	0,3126	0,0562	0,2730	0,0842	0,0703	0,3637	−0,1300	0,2590
	CD11b^+^ cells	0,1815	0,1824	0,4140	0,0159	0,1208	0,2741	−0,1791	0,1858
	CD3^+^ cells	0,2203	0,1348	0,3471	0,0381	0,2718	0,0851	−0,1526	0,2236
	CD11b^−^/CD3^−^ cells	0,2946	0,0679	0,3596	0,0327	−0,2078	0,1491	−0,2313	0,1229

### Melatonergic system regulation by immunological modulators

BM and spleen cells, collected at ZT06 (low levels of melatonin), were stimulated or not with LPS [1 μg/ml] or IL10 [3 and 100 ng/ml] as described in materials and methods.

**Figure 7:**
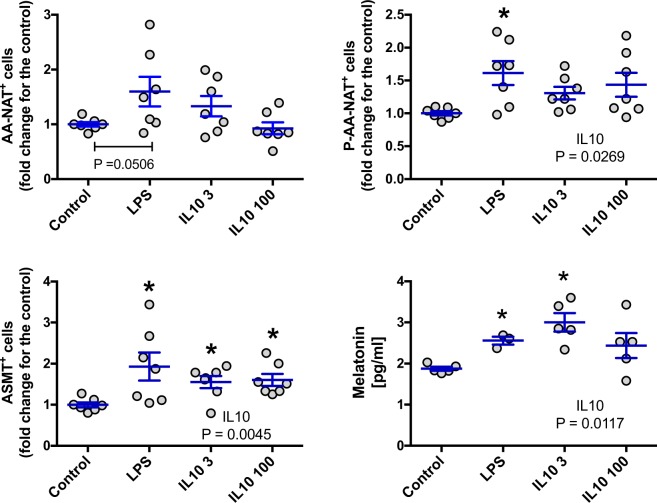
Effect of LPS and IL-10 on the AA-NAT, P-AA-NAT and ASMT expression and melatonin levels in BM cells. Cells were collected at ZT6, plated at 6.67 × 10^6^ cells/mL and stimulated with LPS 1 μg/ml, IL10 3 ng/ml (IL10 3) or IL10 100 ng/ml (IL10 100) for 6 h. After stimulation, cells and supernatant were collected for measurement of the enzyme expression by flow cytometry and for melatonin quantification by ELISA, respectively. Enzyme expression: values are normalized by the enzyme expression of the control. Results are expressed as mean ± SEM, n = 3–7 animals from 2 independent experiments. Control vs LPS: data were analyzed by Student “t” test. Control vs IL10 3 and 100: data were analyzed by one-way ANOVA with Tukey’s post hoc. *P < 0.05 vs control.

**Figure 8:**
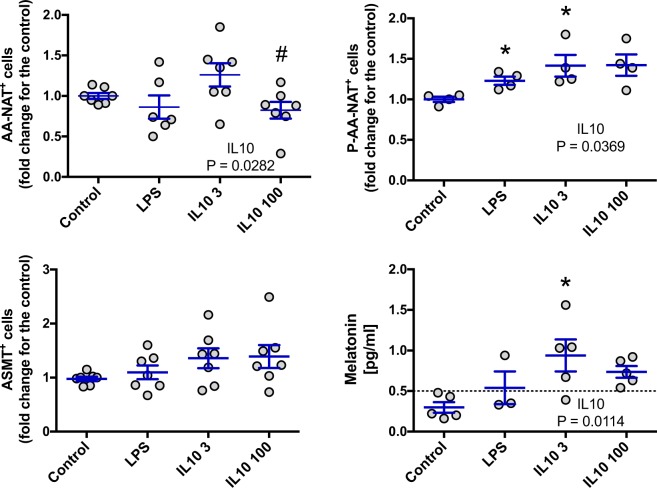
Effect of LPS and IL-10 on the AA-NAT, P-AA-NAT and ASMT expression and melatonin levels in spleen cells. Cells were collected at ZT6, plated at 6.67 × 10^6^ cells/mL and stimulated with LPS 1 μg/ml, IL10 3 ng/ml (IL10 3) or IL10 100 ng/ml (IL10 100) for 6 h. After stimulation, cells and supernatants were collected for measurement of the enzyme expression by flow cytometry and for melatonin quantification by ELISA, respectively. Enzyme expression: values are normalized by the enzyme expression of the control. Results are expressed as mean ± SEM, n = 3–7 animals from 2 independent experiments. Control vs LPS: data were analyzed by Student “t” test. Control vs IL10 3 and 100: data were analyzed by one-way ANOVA with Tukey’s post hoc. *P < 0.05 vs control; ^#^P < 0.05 vs cells treated with IL10 3 ng/ml.

For BM cells, both LPS and IL10 induced an increase in melatonin levels that can be easily explained by an increase in the expression of P-AA-NAT and ASMT. The effect of IL10 on the production of melatonin appears to be dose dependent (Fig. 7). In the case of spleen cells, LPS induced an increase in the expression of P-AA-NAT but, this effect was not sufficient to alter melatonin levels. On the other hand, IL10, increased melatonin in the medium, possibly due to the increase in the expression of AA-NAT and P-AA-NAT. Again, the effect of IL10 was dose dependent (Fig. 8).

## Discussion

Previous studies have shown the presence of the melatonergic biosynthetic pathway enzymes (AA-NAT/P-AA-NAT and ASMT) in extra-pineal tissues and the existence of extra-pineal melatonin synthesis. Here, we characterized the local expression of the melatonergic biosynthetic pathway and the local melatonin levels throughout the day in the BM and in the spleen, two important organs of the immunological system, comprising a hematopoietic tissue and a lymphoid secondary organ, respectively. Our results show that in the BM, the expression of the enzymes varies rhythmically, especially in the CD11b^+^ and CD11b^−^/CD3^−^ cells. In the spleen, the variation of the enzymes is rhythmic only for AA-NAT/P-AA-NAT, these effects were observed in all of the cellular population evaluated. Importantly, the local melatonin levels vary rhythmically in both organs (with higher levels at the night phase).

Although the limited amount of data regarding the daily expression of the melatonergic biosynthetic pathway in extra-pineal tissues, researches have shown that with age, the mRNA expressions and the enzymatic activity of AA-NAT and ASMT changes differentially in the spleen, the spleen, the liver and the heart^24,39^, as well as, the existence of day/night or daily variations in melatonin levels^39,40^. Therefore, the melatonergic biosynthetic pathway is also being regulated in extra-pineal tissues.

In the pineal gland, melatonin production is regulated by the sympathetic nervous system (SNS), where an adrenergic stimulus is necessary to induce the AA-NAT activity. In case of the immune system, it is known that the SNS regulates different rhythmic functions of immunological cells, like specific cellular responses, activation and migration^41–43^. Therefore, it is possible to speculate that the expression of the melatonergic biosynthetic enzymes in immune cells could also be controlled by adrenergic stimulation. Moreover, the existence of feedback between melatonin and clock genes has also been discussed^44,45^. This hypothesis is supported by the presence of sympathetic innervation in BM and spleen^46^, and because adrenergic stimulation induces melatonin production in macrophage cell lines and in BM-derived dendritic cells^19^. In line with that, the noradrenaline-induced TNF peak in the BM, is pivotal for the local rhythmic profile of melatonin^25^. Moreover, considering the dual effects of corticosterona^37^ and the potentiation induced by interferon-gamma on pineal noradrenaline-induced melatonin synthesis^47^, it will be interesting to evaluate the interplay of these immunoregulatory molecules on the daily production of melatonin in the BM and spleen.

In the BM, we found positive and significant correlations between the local melatonin levels and its enzymes, principally marked by the expression of P-AA-NAT in all cellular types evaluated (Fig. 6, Table 1). This data is quite interesting, since the control of the AA-NAT phosphorylation is how the melatonin production is regulated in the pineal. On the other hand, the capacity of the BM to produce melatonin was already showed^23^, and confirmed in pinealectomized animals. In this case, although the circulating melatonin levels significantly decreased, the melatonin content in the BM remained considerably high, without altering the enzymatic activity of local AA-NAT and ASMT^22^, leaving open the possibility that the BM is producing part of that melatonin. In the present work, the profile of the local melatonin levels in the BM is very similar to the profile obtained from the MFIs index (P-AA-NAT + ASMT; with positive correlations), which was significant for the three cellular populations. These data provide further evidences in favor to the idea that the BM could be rhythmically producing its own melatonin, supplementing the one that comes from the pineal gland.

In the case of the spleen, the variation of the melatonergic biosynthetic pathway shows a greater expression during the diurnal phase, at which point a melatonin peak would be expected; but the melatonin peak occurs only in the dark phase, without correlation between melatonin levels and the expression of its enzymes (Fig. 6, Table 1). Therefore, it is most likely that nocturnal melatonin in the spleen is derived from the pineal gland. Even so, one cannot exclude that the spleen is producing melatonin, since studies have shown that, in experiments realized at ZT06, AA-NAT and ASMT present enzymatic activity^24^. Nevertheless, given the low percentage of cells with potential of melatonin production, the low median intensity of fluorescence of those cells (compared with those of the BM) and the enzymatic rhythmic profiles, it is probable that during the light phase, the spleen is producing melatonin in very low concentrations.

To confirm the hypothesis that BM and spleen cells are capable of producing melatonin and that this production is being regulated differently in each organ; we treated cells from both organs *in vitro* with LPS, an immunological activator capable of inducing melatonin synthesis in macrophages^26^; and IL10, an important immunomodulatory cytokine. Melatonin levels were again higher in BM cells, and although IL10 modulated melatonin synthesis in both spleen and BM cells, LPS only had an effect on BM cells. Interestingly, for both organs, all increases of melatonin were related to an increase in the enzyme expression. Showing that stimuli of different nature, such as LPS and IL10, have different effects on the melatonergic system in spleen cells. In the case of IL10, the effects were dose-dependent in both spleen and BM cells.

Different functions of the immune cells like cytokines production^48^, cellular responses, proliferation and migration^49,50^ are controlled by melatonin in a rhythmic way. In this sense, it was recently shown that melatonin is important to synchronize the mature blood cell production and the hematopoietic stem cell repopulation in the BM^25^. Considering the spleen, limited amount of data are available about the effect of the endogen melatonin in specific functions of this organ, but it is know that melatonin affect the activation and differentiation of T cells^51^ and increases the lymphocytic proliferation in different animal models^15,52,53^. Interestingly, melatonin levels and the enzymes, as well as the lymphocytes proliferation, decrease with age in the spleen^52,53^; showing a direct correlation between melatonin and the immune response of the spleen cells. Additionally, melatonin plays a central role in surveillance against infection and inflammatory and recovery phase of acute defense responses^26,54,55^. Taking into account all the above mentioned, this information strengthens the idea that the modulation of local melatonin is tissue and organ specific and these differences are associated not only with the circadian profile of the system but also with the cell responsiveness to immune-related signals.

In conclusion, as many immune cellular functions vary rhythmically, the cellular functions that melatonin exerts are being differentially regulated in each organ and in each cell type in chronobiologic-dependent manners. Therefore, the importance of taking into account the rhythmic profiles of the immune cells in terms of different profiles of the melatonergic biosynthetic pathway expression will provide a better understanding of the physiological role of extra-pineal melatonin production in tissues and cells. In addition, the pioneering data of this work will allow to propose more refined experiments to future researches focused on the modulation of this pathway in the treatment of immunological diseases like hematopoietic tumors and uncontrolled inflammatory conditions, processes that we are increasingly seeing that the time of the day that treatments occurs is crucial to their efficiency.

## Methods

### Animals

Male Wistar rats (8–12 weeks old, 250–300 g), receiving water and food ad libitum, were kept at 22.0 ± 2.0 °C under a 12:12 h light/dark cycle (lights on at 06:00 h = Zeitgeber time zero or ZT0). Animals were killed by decapitation at nine different ZTs (12.25, 15, 18, 21, 23.75, 3, 6, 9, and 11.75) and the pineal gland, BM femurs and the spleen were collected. Animals were obtained from the animal facility of the Department of Physiology - Institute of Bioscience of University of São Paulo (IB-USP, São Paulo, Brazil).

### Ethical approval

All animal protocols were performed in accordance with the ethical standards of the National Council on Experimental Animal Control and were approved by Ethical Committee for Animal Experimentation (license numbers 207/2014 and 253/2016) of the Institute of Bioscience of University of São Paulo.

### Drugs and reagents

Rat interleukin-10 (IL-10) and lipopolysaccharide (LPS, from Escherichia coli serotype 0127:B8) were purchased from Sigma (St Louis, MO, USA). RPMI 1640 medium were purchased from GIBCO (Grand Island, New York, NY, USA). Penicillin/streptomycin was purchased from Life Technologies (Grand Island, NY, USA).

### BM and spleen cell culture

BM cells were obtained from the femur by flushing with 10 ml RPMI 1640 medium supplemented with 100 U/ml penicillin and 0.1 mg/ml streptomycin (RPMI-PS). Spleen cells were obtained by mechanically dispersion using a 100 μm cell strainer filter (Falcon®), washing with 10 ml RPMI-PS. After centrifugation (500 g, 10 min), cells were resuspended in 1 mL RPMI-PS, plated on 24-well plates (2 × 10^6^ cells/well, 500 μl), stimulated or not with LPS [1 μg/ml] or IL10 [3 and 100 ng/ml] and maintained at 37 °C, 5% CO_2_ for 6 h. Supernatant and cells were collected for melatonin quantification by ELISA and for measurement of enzyme expression by flow cytometry, respectively.

### Processing of the samples for enzymes analyses by flow cytometry

BM cells were obtained from the femur by flushing with 1 mL of cold Phosphate-Buffered Saline (PBS; 137 mM NaCl, 2.7 mM KCl, 10 mM Na_2_HPO_4_ and 1.8 mM KH_2_PO_4_). Spleen cells were obtained by mechanically dispersion using a 100 μm cell strainer filter (Falcon®), washing with cold PBS. Cells were fixed with 2% paraformaldehyde (PFA – in PBS, 10 min at 4 °C), permeabilized with 0.1% Tryton X-100 (in PBS, 10 min, room temperature), and blocked with 3% bovine serum albumin (BSA – in PBS, 1 h, room temperature).

### Flow cytometry

Cells were incubated with rabbit anti- AA-NAT (S0564), P-AA-NAT (S0814) (Sigma-Aldrich, St. Louis, MO, USA) or ASMT (IM-044, Imuny Biotechnology, São Paulo, Brazil) primary antibodies (1/400, 30 min, room temperature, 3% BSA in PBS). After, cells were washing with PBS and labeled with a mix containing goat anti-rabbit PE-Cy5-conjugated IgG (SC3844, Santa Cruz Biotechnology, Santa Cruz, CA, USA), mouse anti-rat FITC-conjugated CD11b (BD554982, Biosciences, San Jose, CA, USA) and mouse anti-rat PE-conjugated CD3 (Biolegend 201412, San Diego, CA, USA) antibodies (1/400, 30 min, room temperature, 3% BSA in PBS). Samples were acquired with an Amnis® FlowSight® (Luminex, Austin, TX, USA) and data were analyzed with IDEAS® software.

### Melatonin quantification by ELISA

The content of melatonin in BM was obtained from the femur by flushing with 1 ml of cold PBS, the fluid was centrifuged (14000 g, 5 min at 4 °C) and supernatant was collected. For the spleen melatonin, half spleen was homogenized in 2 mM Tris-HCl buffer (adding 1 mM EDTA and 1 mM EGTA) in a ratio of 130 mg of tissue to 200 μl of Tris-HCl buffer; the homogenate was then centrifuged (14000 g, 5 min at 4 °C) and the supernatant was collected.

Melatonin was measured using ELISA kits following the manufacturer’s instructions (Immuno Biological Laboratories, Hamburg, Germany). The detection limits of the melatonin ELISA kits were 0.5 pg/ml. Values were relativized to the amount of protein, as measured by the Bradford colorimetric method (BioRad, Hercules, CA, USA).

### Melatonin quantification in the pineal gland by HPLC

Melatonin contents in the pineal glands were determined by HPLC (high-performance liquid chromatography) with electrochemical detection as previously described^56^. Briefly, the glands were homogenized in ice-cold 0.1 M perchloric acid (120 μl) containing 0.02% EDTA and 0.02% sodium bisulfite, centrifuged (13000 g, 5 min at 4 °C), and 20 μl of the supernatant was injected into the chromatographic system (Waters, Milford, MA, USA), which was isocratically operated with the mobile phase consisting of 0.1 mM sodium acetate, 0.1 mM citric acid, 0.15 mM EDTA, 25% methanol, pH 3.7 at a flow rate of 0.50 ml/min, through a 5-mm Resolve C-18 reversed-phase column (Waters, Mildford, MA, USA). The detector potential was adjusted to +0.90 V versus Ag/AgCl reference electrode. The detection limit was 500 pg/injection.

### Statistical analysis

Data were expressed as mean ± SEM. Time series were analyzed using the Fourier and Cosinor analysis (Chronobiology Software El Temps, ©Antoni Díez-Noguera, Barcelona, CA, Spain). Comparisons were performed using One-way Analysis of Variance (ANOVA) followed by Tukey’s post hoc test to see the time of maximal and/or minimal expressions and for the *in vitro* treatments with IL10. Student “t” test was used to compare the *in vitro* treatments with LPS. We used Pearson’s correlation to evaluate the association between enzymes levels and the content of melatonin detected. P < 0.05 were considered statistically significant. Analyses were performed using GraphPad Prism 7.0 and R (https://www.r-project.org).

## Supplementary information

Supplementary information.
