# Supplementary material for: Rhythmic expression of the melatonergic biosynthetic pathway and its differential modulation in vitro by LPS and IL10 in bone marrow and spleen
Source: Sci Rep. 2020 Mar 16;10:4799. doi: 10.1038/s41598-020-61652-5 (PMC7075864; doi:10.1038/s41598-020-61652-5)
Supplement: Supplementary file 1 — Supplementary information. [file 41598_2020_61652_MOESM1_ESM.docx]

***Rhythmic expression of the melatonergic*** ***biosynthetic pathway and its differential modulation in vitro by LPS and IL10 in bone marrow and spleen.***

**Marlina O. Córdoba-Moreno^1*^, Ewerton Da Silva de Souza^2^, Caroline L. Quiles^2^, Débora Dos Santos Silva^1^, Gabriela S. Kinker^1^, Sandra M. Muxel^3^, Regina P. Markus^2^, Pedro A. Fernandes^1*^**

^1^Laboratory of Neuroimmunoendocrinology, Department of Physiology, Institute of Bioscience, University of São Paulo, 05508-900 São Paulo, Brazil

^2^Laboratory of Chronopharmacology, Department of Physiology, Institute of Bioscience, University of São Paulo, 05508-900 São Paulo, Brazil

^3^Laboratory of Trypanosomatid Physiology, Department of Physiology, Institute of Bioscience, University of São Paulo, 05508-900 São Paulo, Brazil

* Correspondence: marlinaocm@usp.br (MOCM); pacmf@usp.br (PAF); Tel.: +55-11-3091-7612

**Supplementary material**

**Tables**

Table 1. Cosinor analysis of the melatonin levels and the percentage of CD11b^+^, CD3^+^ and CD11b^-^/CD3^-^ cells in the bone marrow (BM) and the spleen. Cosinor analysis was evaluated in harmonics (nh) one (24 hours rhythm) and four (six hours rhythm).

|  | nh | Melatonin | | CD11b^+^ | | CD3^+^ | | CD11b^-^/CD3^-^ | |
| --- | --- | --- | --- | --- | --- | --- | --- | --- | --- |
|  |  | Power | P-value | Power | P-value | Power | P-value | Power | P-value |
| BM | 1 | 31,3 | 0,0004 | - | - | 16,34 | 0,314 | 62,15 | 0,0001 |
|  | 4 | 39,76 | 0,0001 | - | - | 1,05 | 0,9209 | 1,75 | 0,5468 |
| Spleen | 1 | 57,87 | 0 | 51,83 | 0,0001 | 31,85 | 0,0093 | 26,62 | 0,0030 |
|  | 4 | 10,9 | 0,0068 | 3,94 | 0,2045 | 10,59 | 0,1319 | 13,71 | 0,0244 |

Table 2. Cosinor analysis of the expression of the melatonergic biosynthetic pathway enzymes in the bone marrow (BM) and the spleen cells. Cosinor analysis was evaluated in harmonics (nh) one (24 hours rhythm) and four (six hours rhythm).

|  | | nh | Total cells | | | | CD11b^+^ cells | | | | CD3^+^ cells | | | | CD11b^-^/CD3^-^ cells | | | |
| --- | --- | --- | --- | --- | --- | --- | --- | --- | --- | --- | --- | --- | --- | --- | --- | --- | --- | --- |
|  |  |  | %Cells | | MFI | | %Cells | | MFI | | %Cells | | MFI | | %Cells | | MFI | |
|  |  |  | Power | P-value | Power | P-value | Power | P-value | Power | P-value | Power | P-value | Power | P-value | Power | P-value | Power | P-value |
| BM | AA-NAT | 1 | 42,39 | 0,0025 | 10,01 | 0,5194 | 15,68 | 0,1993 | 10,01 | 0,5194 | 24,21 | 0,0823 | 6,32 | 0,5383 | 8,56 | 0,4859 | 5,29 | 0,4824 |
|  |  | 4 | 0,34 | 0,9181 | 0,38 | 0,974 | 8,47 | 0,395 | 0,38 | 0,974 | 1,15 | 0,8619 | 8,31 | 0,449 | 9,41 | 0,4546 | 48,8 | 0,0100 |
|  | P-AA-NAT | 1 | 27,23 | 0,0005 | 5,9 | 0,0687 | 6,38 | 0,5187 | 21,2 | 0,0073 | 19,57 | 0,0624 | 7,54 | 0,396 | 31,17 | 0,0047 | 4,79 | 0,2086 |
|  |  | 4 | 1,11 | 0,527 | 29,59 | 0,0004 | 2,37 | 0,7765 | 15,72 | 0,0182 | 14,14 | 0,1184 | 9,84 | 0,3074 | 0,6 | 0,8418 | 28,6 | 0,0025 |
|  | ASMT | 1 | 9,41 | 0,0464 | 7,32 | 0,313 | 21,31 | 0,175 | 22,62 | 0,0007 | 36,18 | 0,037 | 5,02 | 0,4192 | 18,57 | 0,1622 | 10,92 | 0,2033 |
|  |  | 4 | 13,48 | 0,0182 | 31,07 | 0,0222 | 3,36 | 0,7307 | 20,15 | 0,0011 | 14,03 | 0,2188 | 7,52 | 0,2847 | 9,21 | 0,3763 | 37,27 | 0,015 |
|  |  |  |  |  |  |  |  |  |  |  |  |  |  |  |  |  |  |  |
| Spleen | AA-NAT | 1 | 32,25 | 0,0441 | 11,37 | 0,0466 | 17,26 | 0,177 | 18,68 | 0,0299 | 8,42 | 0,5666 | 19,87 | 0,1489 | 8,1 | 0,4625 | 7,67 | 0,5751 |
|  |  | 4 | 0,25 | 0,9686 | 5,06 | 0,2069 | 2,89 | 0,7188 | 4,55 | 0,3384 | 5 | 0,7087 | 2,18 | 1 | 10,87 | 0,3636 | 2,85 | 1 |
|  | P-AA-NAT | 1 | 60,74 | 0,0017 | 34,13 | 0,0889 | 39,68 | 0,007 | 32,51 | 0,0311 | 49,13 | 0,0104 | 33,34 | 0,0499 | 55,17 | 0,0003 | 48,96 | 0,0174 |
|  |  | 4 | 0,22 | 0,9572 | 0,4 | 1 | 20,21 | 0,0469 | 7,38 | 0,3673 | 0,87 | 0,8831 | 4,56 | 1 | 5,64 | 0,198 | 4,27 | 1 |
|  | ASMT | 1 | 24,27 | 0,1983 | 0,21 | 0,9825 | 3,05 | 0,8131 | 1,38 | 0,9161 | 36,51 | 0,0548 | 16,59 | 0,2462 | 6,87 | 0,5131 | 24,48 | 0,2335 |
|  |  | 4 | 5,82 | 0,6483 | 4,74 | 1 | 1,8 | 0,884 | 2,84 | 1 | 1,95 | 0,8186 | 3,48 | 1 | 11,86 | 0,3307 | 2,84 | 1 |

**Figure**

**Figure 1.** Predictive index profile versus melatonin levels. The predictive index was constructing by summing the frequency of cells (left panel) or by summing the Median fluorescence of intensity (MFI) (right panel) that express the melatonergic enzymes (P-AA-NAT and ASMT) in each cellular population in the bone marrow and the spleen.
